# Relapsed Medulloblastoma in Pre-Irradiated Patients: Current Practice for Diagnostics and Treatment

**DOI:** 10.3390/cancers14010126

**Published:** 2021-12-28

**Authors:** Rebecca M. Hill, Sabine L. A. Plasschaert, Beate Timmermann, Christelle Dufour, Kristian Aquilina, Shivaram Avula, Laura Donovan, Maarten Lequin, Torsten Pietsch, Ulrich Thomale, Stephan Tippelt, Pieter Wesseling, Stefan Rutkowski, Steven C. Clifford, Stefan M. Pfister, Simon Bailey, Gudrun Fleischhack

**Affiliations:** 1 Wolfson Childhood Cancer Research Centre, Newcastle University Centre for Cancer, Newcastle upon Tyne NE1 7RU, UK; Steve.Clifford@newcastle.ac.uk (S.C.C.); Simon.Bailey@newcastle.ac.uk (S.B.); 2 Princess Máxima Center for Pediatric Oncology, 3584 CS Utrecht, The Netherlands; S.L.A.Plasschaert-2@prinsesmaximacentrum.nl (S.L.A.P.); m.h.lequin@umcutrecht.nl (M.L.); P.Wesseling-2@prinsesmaximacentrum.nl (P.W.); 3 Department of Particle Therapy, West German Proton Therapy Centre Essen (WPE), West German Cancer Center (WTZ), University Hospital Essen, 45147 Essen, Germany; beate.timmermann@uk-essen.de; 4 Department of Pediatric and Adolescent Oncology, Gustave Roussy, 94800 Villejuif, France; Christelle.DUFOUR@gustaveroussy.fr; 5 Department of Neurosurgery, Great Ormond Street Hospital, London WC1N 3JH, UK; Kristian.Aquilina@gosh.nhs.uk; 6 Department of Radiology, Alder Hey Children’s NHS Foundation Trust, Liverpool L12 2AP, UK; Shivaram.Avula@alderhey.nhs.uk; 7 UCL Great Ormond Street Institute of Child Health, London WC1N 1EH, UK; l.k.donovan@ucl.ac.uk; 8 Institute of Neuropathology, DGNN Brain Tumor Reference Center, University of Bonn, 53127 Bonn, Germany; torsten.pietsch@ukbonn.de; 9 Department of Neurosurgery, Charité-Universitätsmedizin Berlin, 13353 Berlin, Germany; ulrich-wilhelm.thomale@charite.de; 10 Department of Pediatrics III, Center for Translational Neuro- and Behavioral Sciences (CTNBS), University Hospital of Essen, 45147 Essen, Germany; stephan.tippelt@uk-essen.de; 11 Department of Pathology, Amsterdam University Medical Centers/VUmc, 1081 HV Amsterdam, The Netherlands; 12 Department of Pediatric Hematology and Oncology, University Medical Center Hamburg-Eppendorf, 20246 Hamburg, Germany; s.rutkowski@uke.de; 13 Hopp Children’s Cancer Center Heidelberg (KiTZ), 69120 Heidelberg, Germany; s.pfister@kitz-heidelberg.de; 14 Division of Pediatric Neurooncology, German Cancer Research Center (DKFZ), 69120 Heidelberg, Germany; 15 Department of Pediatric Oncology and Hematology, Heidelberg University Hospital, 69120 Heidelberg, Germany

**Keywords:** medulloblastoma, relapse

## Abstract

**Simple Summary:**

Medulloblastoma is the commonest malignant brain tumour of childhood. Disease relapse following maximal multi-modal therapy including upfront craniospinal irradiation (CSI) is almost always fatal and occurs in approximately 30% of patients. Importantly, patients rarely die of other causes, and consequently relapsed medulloblastoma (rMB) accounts for 10% of all childhood cancer deaths. There is a variety of life-prolonging treatment options at relapse including neurosurgery, re-irradiation, early-phase trials, and chemotherapy. Here, we summarise best practice for investigations including re-biopsy and molecular characterisation of rMB, treatment decision making, and available treatment options. We also highlight advances in the understanding of rMB disease biology and prognostic factors, and look towards future developments such as targeted therapies, liquid biopsies and disease modelling strategies to improve outcomes in this area of unmet clinical need.

**Abstract:**

Relapsed medulloblastoma (rMB) accounts for a considerable, and disproportionate amount of childhood cancer deaths. Recent advances have gone someway to characterising disease biology at relapse including second malignancies that often cannot be distinguished from relapse on imaging alone. Furthermore, there are now multiple international early-phase trials exploring drug–target matches across a range of high-risk/relapsed paediatric tumours. Despite these advances, treatment at relapse in pre-irradiated patients is typically non-curative and focuses on providing life-prolonging and symptom-modifying care that is tailored to the needs and wishes of the individual and their family. Here, we describe the current understanding of prognostic factors at disease relapse such as principal molecular group, adverse molecular biology, and timing of relapse. We provide an overview of the clinical diagnostic process including signs and symptoms, staging investigations, and molecular pathology, followed by a summary of treatment modalities and considerations. Finally, we summarise future directions to progress understanding of treatment resistance and the biological mechanisms underpinning early therapy-refractory and relapsed disease. These initiatives include development of comprehensive and collaborative molecular profiling approaches at relapse, liquid biopsies such as cerebrospinal fluid (CSF) as a biomarker of minimal residual disease (MRD), modelling strategies, and the use of primary tumour material for real-time drug screening approaches.

## 1. Introduction

Relapsed medulloblastoma (rMB) occurs in approximately 30% of all patients diagnosed with the disease. This is despite intensive multi-modal standard upfront treatment comprising maximal safe resection, craniospinal irradiation (CSI) and chemotherapy. Prognosis following relapse is dismal, with less than 5% of patients surviving; disease relapse is therefore the most adverse prognostic factor in MB. Furthermore, patients diagnosed with MB rarely die of other causes; less than 2% of patients who relapse die of treatment complications (Hill et al., 2020, unpublished data). Consequently, rMB accounts for a considerable, and disproportionate amount of childhood cancer deaths (~10%) and remains an urgent area of clinical unmet need [1,2,3,4,5,6,7,8].

Research efforts in the recent past have predominantly focused on the disease at diagnosis, in part due to the more readily available biopsy samples and annotated clinical trials. This has led to the discovery of four principal MB molecular groups (MB_WNT_, Wnt/wingless-activated; MB_SHH_, Sonic-hedgehog-activated; MB_Group3_, Group3; and MB_Group4_, Group4), second-generation subgroups, genomic characteristics and their clinical relevance [9,10,11]. The definitions and terminology of these molecular groups and subgroups have now been harmonised and are being included in the 5th edition of the WHO classification of CNS tumours [12]. Lately, there have been an increasing number of studies focused on rMB, alongside international efforts such as SMPaeds, INFORM, MAPPYACTS, eSMART, MATCH and PRISM, which sample, molecularly characterise, and explore biologically informed drug–target matches across a range of high-risk/relapsed paediatric tumours [1,2,3,6,13,14,15,16,17,18]. Combined, these studies provide an increasingly detailed overview of rMB, including first insights into disease evolution and its potential role in treatment failure. They also provide evidence that a relevant proportion of suspected rMB, upon re-biopsy and molecular pathology assessment, are second malignancies, typically radiation-induced high-grade gliomas with a fundamentally different disease biology [19].

Here, we provide a compendium of current understanding for rMB in pre-irradiated patients, i.e., children who received upfront CSI at initial diagnosis. We focus on prognostic factors, the clinical diagnostic process, best practice for treatment decision making, and look towards future developments to improve outcomes.

## 2. Prognostic Factors

Multiple studies over the last decade have confirmed that in the majority of rMB, principal molecular group (MB_WNT_, MB_SHH_, MB_Group3_ or MB_Group4_) is conserved between diagnosis and relapse [1,3,6,13,20]. Novel molecular subgroups such as subgroups 1–8 in MB_Group3/4_ rarely show variation between diagnosis and relapse but numbers are small and the significance of this finding unclear [6,10,13].

The timing of disease relapse is molecular group dependent. Several independent studies have shown that MB_Group4_ displays a more indolent course, with either a prolonged time to relapse or time to death post-relapse [2,3,6,13]. Conversely, MB_Group3_ has a rapid disease course (time to relapse or time to death post-relapse) [2,6,13]. Time to relapse is also associated with time to death post-relapse, i.e., patients with a prolonged time to relapse tend to have a prolonged time to death [5,13,21]. Patterns of disease relapse are also associated with molecular group and have prognostic relevance. Typically, both MB_Group3_ and MB_Group4_ relapse in metastatic locations, with or without concurrent local disease in the posterior fossa. MB_SHH_ relapses more frequently in the posterior fossa when compared to MB_Group3_ and MB_Group4_; however, the predominant pattern of relapse in MB_SHH_ varies between studies (i.e., local versus metastatic) [2,3,13,21]. Recent reports show that patients who have either isolated posterior fossa relapses or nodular disease (single or multiple discrete lesions on MRI) have prolonged survival post-relapse [2,4]. However, the ability to understand this finding in the context of local treatment decisions (e.g., re-resection and focal radiotherapy) is challenging and this finding should be interpreted with caution.

Initial studies which have examined the molecular landscape of rMB identify varying amounts of shared biology and tumour evolution between diagnoses and relapse [1,6,13,20]. Common findings include early reports of mutations within *TP53* as an enriched/acquired event in rMB [1,20]. Subsequent studies also identify aberrations in key pathways at relapse including chromatin modification and DNA damage response [6,13]. Importantly, the putative genetic drivers in rMB identified to date are frequently maintained, and thus remain actionable throughout the disease course. However, approximately 30–60% of rMB drivers are found at relapse only. The incidence of emergent rMB driver varies significantly between molecular groups, but crucially these discoveries highlight the importance of re-biopsy and molecular characterisation at both diagnosis and relapse [1,6,13].

In summary, a small number of studies have reported both shared biology between diagnosis and relapse, and tumour evolution with emergent drivers demonstrated at relapse. These initial discoveries highlight the importance of further study of disease biology at relapse. However, to advance these findings, a more collaborative infrastructure nationally and internationally, across both the clinical and research disciplines, is required to collect and utilise these rare samples to their maximal potential [22].

## 3. Clinical Diagnostic Process

### 3.1. Signs and Symptoms

**Figure 1:**
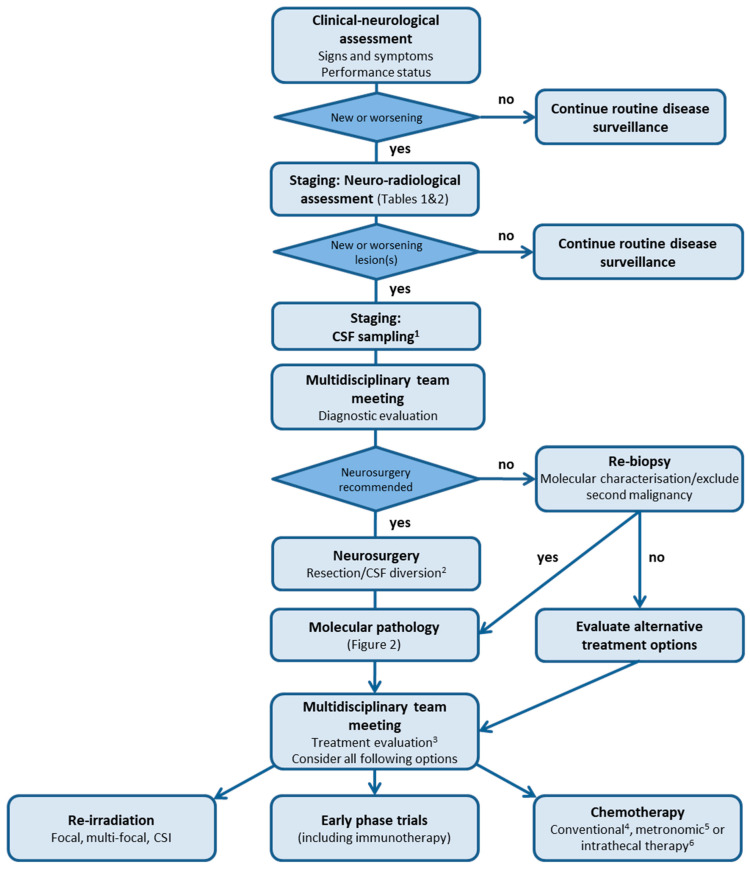
Flowsheet representing the clinical diagnostic process and treatment options for relapsed medulloblastoma. ^1^ Lumbar CSF if no clinical contraindication and additionally ventricular CSF if Ommaya or Rickham Reservoir available. CSF staging can occur ≥15 days after neurosurgery if recommended. ^2^ Re-biospy can be performed at the time of CSF diversion if technically feasible. ^3^ Treatment evaluation can be undertaken at the same time as diagnostic evaluation if, for example, neurosurgery is not being undertaken. Consider intensity and toxicities of previous treatment, time from initial treatment, and clinical status. ^4^ Consider temozolomide monotherapy, TOTEM, and TEMIRI ± bevacizumab. ^5^ Consider MEMMAT, modified MEMMAT or COMBAT regimen. ^6^ Consider either etoposide, toptecan or cytarabine when there is leptomeningeal disease. CSF, cerebrospinal fluid; CSI, craniospinal irradiation.

The clinical picture often differs at relapse when compared to initial tumour diagnosis, where signs of raised intracranial pressure (headaches, nausea, vomiting, fatigue, and double vision) and tumour-related symptoms such ataxia are observed. Detection of asymptomatic rMB can occur through surveillance imaging (Figure 1). This is particularly noted in the HIT-SIOP-PNET4 standard-risk MB trial, whereby 12/18 (67%) rMB confined to the spinal axis were identified on routine surveillance imaging [4,23]. Conversely, patients can present with worsening of known or new neurological deficits dependent on the site and size of the lesion(s), and the degree of leptomeningeal spread of disease [8]. Sudden-onset symptoms of raised intracranial pressure are typically associated with a rapidly growing local relapse in the posterior fossa or with pronounced leptomeningeal dissemination. These patients require neurological monitoring and urgent imaging (MRI or CT evaluation). Cranial nerve (CN) impairment is also observed in approximately 25% of symptomatic patients (unpublished data HIT-REZ-97-study cohort) and is either caused by raised intracranial pressure (CN III, IV, and VI), suprasellar disease (CN II) or brainstem/medulla oblongata involvement (CN VIII, IX, X, and XII).

Cerebellar signs such as ataxia, coordination problems, nystagmus, intention tremor, and dizziness are often observed in local rMB. Epileptic seizures, sensory disturbances, neuropsychiatric/cognitive or memory disorders, new neuroendocrinological problems, or unspecific symptoms such as cachexia, weight loss, sleeping problems, and fatigue are rare at relapse, occurring in less than 10% of symptomatic patients (unpublished data HIT-REZ-97-study cohort). Extra-central nervous system (CNS) relapses are rare and more frequently observed in adult patients, with bone and/or bone marrow involvement leading to bone pain and cytopenias [24].

### 3.2. Staging: Neuro-Radiological Assessment

MRI is the mainstay of radiological assessment and the standard imaging protocol for paediatric brain tumour studies has been published based on consensus by the SIOPE Brain Tumour Imaging Group [25]. The Response Assessment in Paediatric Neuro-Oncology (RAPNO) committee have also published recommendations for imaging in MB [26]. These two guidelines are highly aligned, with the only difference being the timing of the post-contrast T_2_ FLAIR sequence, which is recommended following gadolinium contrast on the RAPNO guideline, a particularly useful sequence for identifying leptomeningeal metastasis [27,28]. The post-contrast T_2_ FLAIR is optional on the SIOPE protocol. There is flexibility to suit the practice of all centres but, most importantly, the imaging protocol should be consistent for every patient on consecutive scans to enable comparison.

**Figure 2:**
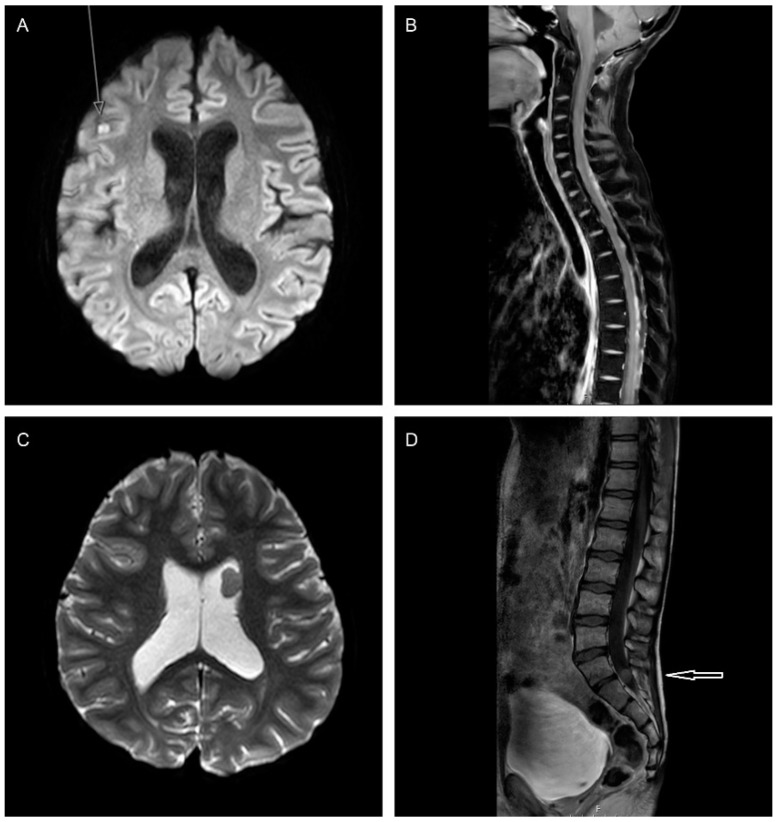
MRI images demonstrating varying patterns of disease relapse in medulloblastoma. (**A**,**B**). A 15-year-old patient with a non-metastatic MB_Group4_ at diagnosis, treated initially with CSI and chemotherapy. Six months following the end of treatment, leptomeningeal metastases are detected in the right cerebral hemisphere ((**A**), axial b 1000) and spine ((**B**), post-contrast sagittal T_1_W DIXON). (**C**). An 11-year-old patient with a non-metastatic MB_Group4_ at diagnosis, treated initially with CSI and chemotherapy. Three years following end of treatment, an intraventricular metastasis is discovered in the left frontal horn (axial ADC map). (**D**). A 14-year-old patient with a non-metastatic MB_Group4_ at diagnosis, treated with CSI and chemotherapy. Two years following CSI, an intraspinal relapse at S1–S2 is noted (post-contrast sagittal T_1_W DIXON).

Given the high likelihood of a metastatic relapse (Figure 2), it is essential to image the entire neuroaxis (Table 1 and Table 2). There is, however, no evidence for the frequency of spinal imaging during surveillance scanning. When considering rMB, diffusion-weighted imaging (DWI), which differentiates between lesions of low and high cellularity, is of particular importance and can aid in identifying intracranial metastases (Table 1). We also highly recommend including the posterior fossa in the field of view for sagittal spinal imaging. This will provide a late contrast enhanced scan, improving detection of both local relapse and small metastases within the posterior fossa [29,30]. In addition, a balanced steady-state-free precession sequence such as Constructive Interference in Steady State (CISS), Fast Imaging Employing Steady-state Acquisition (FIESTA), T_2_ DRIVE and balanced Fast Field Echo (bFFE), which provide heavily T_2_ weighted images, can be used to identify small drop metastasis in the spinal canal [31]. Some radiologists may suggest DWI for the spinal axis [32]. However, the sequence is very sensitive to field inhomogeneity, frequently seen in spinal imaging leading to artefacts. This is especially noted when using a sagittal DWI sequence, where the slice thickness of the commonly used DWI sequence is larger than that used in the T_1_ post-gadolinium sequence. Furthermore, it has been shown that gadolinium can influence DWI of the brain and may similarly affect DWI of the spine [33].

Finally, rMB can present in a non-CNS site such as bone [24]. This is often identified on imaging the symptomatic site as guided by clinical findings. When a wide metastatic relapse is suspected, a whole-body Short Tau Inversion Recovery (STIR) MRI sequence is recommended as a screening sequence to detect non-CNS sites of disease. In individual cases, imaging with 8F-fluorodeoxyglucose (18F-FDG) Positron Emission Tomography (PET) has also been shown to be helpful in the detection and follow-up of bone and bone marrow metastases [34,35,36].

#### Response Assessment following Relapse

Re-irradiation is increasingly utilised for rMB (see Re-Irradiation). Photon and proton beam therapy (PBT) may induce swelling or enhancement of the treated areas; differentiation therefore between true progression, pseudo-progression and lack of treatment response can be challenging. Unfortunately, there is no MRI sequence that is 100% specific in identifying pseudo-progression, but a combination of structural and multi-modal imaging may be of help [37]. Comparison with the MRI characteristics of the original tumour including its diffusion, spectroscopy and perfusion characteristics either using dynamic susceptibility contrast (DSC) or arterial spin labelling sequences (ASL) is also very important in judging disease response. If the original diagnostic imaging of the tumour was very basic, however, it is harder to differentiate between progression and pseudo-progression. Correlation with clinical status and early follow-up imaging aids decision making and determining whether progressive imaging changes are genuine.

### 3.3. Staging: CSF Sampling

Leptomeningeal disease (LMD) is detectable in approximately 50–80% of patients at relapse [2,4,8,38]. Ideally, both CSF cytology combined with imaging of the neuroaxis should be utilised for more sensitive detection of LMD [26,39]. Recommended standard of care is to perform cytology from lumbar CSF taken ≥15 days after tumour surgery, following spinal MRI to avoid imaging artefacts caused by the procedure, and ideally prior to adjuvant therapy. However, risk factors, such as raised intracranial pressure, alongside the added value of this information in, for example, a confirmed M3 relapse, must always be considered in the context of undertaking a painful procedure.

The presence of clusters of cells showing nuclear atypia such as enlargement, prominent nucleoli, moulding or mitotic activity, and positivity for Neuron-Specific Enolase (NSE) are considered key features for the confident diagnosis of LMD. Not infrequently, however, and even after performing additional immunocytochemistry such as synaptophysin, CSF cytology findings are considered as only ‘suspicious’ and do not allow for definitive staging [40,41,42]. Furthermore, early post-operative or intraoperative CSF sampling might reveal false-positive results due to post-/intraoperative changes with cytological detection of floating ependymal cells, blood cells and tumour cells. Early negative results, however, can be accepted [26].

If an intraventricular device is available (Ommaya or Rickham Reservoir), an additional CSF sample can be taken from this site to increase diagnostic accuracy [43,44]. Recommendation from the RAPNO committee is to take a minimum CSF volume of 2.5 mL in patients younger than 5 years of age and larger volumes in older children and adults [26]. CSF samples should be processed immediately to avoid cytolysis and false-negative results, and 5–10 cytospins should be prepared for assessment [26,39]. For response assessment, in patients with proven LMD and positive CSF cytology, ideally 2 negative CSF samples taken at least 2 weeks apart are considered a response [44]. CSF sampling, however, is not always achievable as a form of response assessment and the risks and benefits of additional procedures should be considered on an individual patient basis.

In summary, lumbar CSF sampling is recommended for staging and, where appropriate, response assessment in rMB. However, at every time point of assessment the contraindications (i.e., raised intracranial pressure), risks (e.g., bleeding and infection), and benefits of this approach must be considered, particularly in paediatric patients where general anaesthesia is frequently required for CSF sampling [26,45].

### 3.4. Molecular Pathology

MBs are highly cellular tumours characterised by densely packed, poorly differentiated neural cells with high mitotic activity and apoptosis. Four histologically entities are recognised—classic (CLA), desmoplastic/nodular (D/N), MB with extensive nodularity (MBEN), and large cell/anaplastic (LC/A)—each with their own clinical associations [46,47,48,49]. Typically, histology remains stable between diagnosis and relapse, although there are occasional reports of acquisition of high-risk features such as LC/A [1].

The four principal molecular groups are now well defined according to transcriptomic and epigenetic analyses and were first recognised in the revised 4th edition of the World Health Organisation (WHO) classification of CNS tumours. MB_SHH_ was further subdivided in this edition according to *TP53* mutation status, and the less well-defined entities of MB_Group3_ and MB_Group4_ combined (non-WNT/non-SHH) [9,50,51,52]. Importantly, at diagnosis, the molecularly defined principal groups demonstrate key clinicopathological and survival associations [9,53]. In the 5th edition of the WHO CNS tumour classification, stratification of MB is unchanged [12]. However, based on the profiling of large numbers of tumours, we now recognise further refinement within the principal molecular groups with four MB_SHH_ subgroups and eight MB_Group3/4_ subgroups [10,11,54,55,56,57]. Many of these second-generation subgroups have clinical utility at diagnosis; for example, subgroup 2 and 3 of MB_Group3/4_ are associated with *MYC* amplification and demonstrate a particularly poor prognosis [10]. The molecular group at diagnosis alongside additional aberrations detected at relapse have demonstrated clinical utility in predicting the nature and timing of relapse and subsequent disease course (see Prognostic Factors).

**Figure 3:**
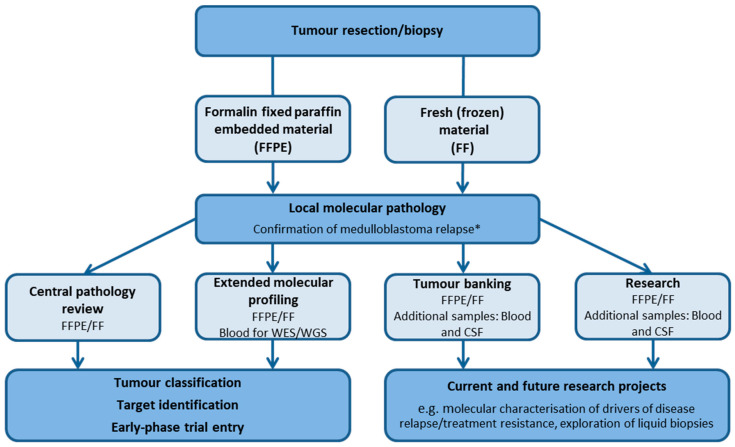
Schematic representing sample considerations following neurosurgical resection/biopsy at relapse. * Confirmation of relapse requires central pathology review and molecular profiling such as DNA methylation array as part of a central review process according to national set-up. WES, whole-exome sequencing; WGS, whole-genome sequencing; CSF, cerebrospinal fluid.

An integrated approach, combining results of morphological and molecular analysis (Figure 3), is recommended for providing the accurate diagnosis of MB whether this be at initial diagnosis or at relapse [58,59]. Tumours can be assigned to their principal molecular group using a combination of immunohistochemical markers (β-catenin, Yap1, p75-NGFR, and Otx2), and targeted sequencing (e.g., *CTNNB1* in MB_WNT_ and *TP53* in MB_SHH_). Additional methods such as fluorescence in situ hybridisation (FISH) for *MYC*/*MYCN* amplification serve as a tool for further risk stratification. DNA methylation profiling adds another layer of information and is a robust and powerful diagnostic tool which can also exclude other histological entities such as second malignancies at relapse [19,60,61,62,63].

Finally, it is important to note that MB may occur in the setting of several inherited cancer syndromes. Germline mutations can occur in *SUFU*, *PTCH1, and ELP1* (naevoid basal cell carcinoma syndrome/Gorlin syndrome), *TP53* (Li–Fraumeni syndrome), *APC* (familial adenomatous polyposis), *CREBBP* (Rubinstein–Taybi syndrome), *NBS1* (Nijmegen breakage syndrome), *PALB2*, *GPR161,* and *BRCA2*, among others [64,65,66,67,68,69,70,71]. Germline mutations are relatively common in patients with MB_SHH_, and this population of patients should be offered genetic counselling at the time of initial diagnosis prior to the onset of radiotherapy or consideration of a clinical trial [70]. Importantly, germline mutations may only be discovered at relapse; genetic counselling should still be offered in this context given the wider implications for family members. When considering an inherited cancer syndrome, treatment at relapse should be balanced against the underlying prognosis of rMB and follow the same principles as other patients (see Intention of Treatment).

## 4. Intention of Treatment

Communication of prognosis and setting of expectations are key to the management of patients with rMB and facilitate joint decision making between patients, families and carers. As our understanding of rMB expands, prognostically relevant information can be utilised to inform these early discussions and treatment choices. For example, an early relapse within the first 12 months of initial treatment completion is highly likely to rapidly progress, especially in the presence of known adverse molecular features such as *TP53* mutation or MB_Group3_ (see Prognostic Factors) [1,2,21]. This knowledge, alongside intensity of previous treatment, time from initial treatment, and clinical status will influence entry into early-phase trials, patient/family choice, and clinician recommendations. Individual patient/family choice must always be taken into consideration, and treatment tailored accordingly.

Here, we discuss the most frequently used and/or evidenced treatment options for patients who relapse with MB following standard multi-modal therapy (Figure 1). While we offer several treatment options, they are typically non-curative and furthermore, may not always be disease-modifying. The choice to either not commence or discontinue ‘active’ treatment and provide symptom-modifying treatment as best supportive care only should always be considered and openly discussed.

## 5. Treatment Modalities and Considerations

### 5.1. Neurosurgery

As previously described, the majority of relapses occur at metastatic sites (Figure 2) with or without disease in the posterior fossa, limiting the frequency of surgery and the ability to assess its role in prolonging survival [2,3,4,13]. Some retrospective reports have suggested that isolated/nodular relapses are associated with prolonged survival [2,4]. In the study undertaken by Sabel et al., surgery in patients with isolated recurrences (11/25) was significantly associated with prolonged survival [4]. However, small patient numbers, heterogeneous treatment approaches and an understandable tendency to restrict care to palliative options preclude a clear understanding of the impact of surgery in rMB. Indeed, many reports have either not investigated, or not demonstrated the benefit of surgery at relapse [3,5,8,21,72].

With limited evidence, a pragmatic approach must be adopted when considering surgery for rMB. There are three overarching situations where surgery may be considered: resection of an isolated nodular lesion, re-biopsy for the purpose of detailed pathological (including exclusion of second malignancy) and molecular characterisation (Figure 3), and palliative diversion of CSF to enhance quality of life.

Complete resection of an isolated lesion, or surgical debulking of a mass lesion should be considered to avoid or improve neurological function such as spinal cord compression. As already described, there is also a potential survival benefit in this scenario, whereby overall survival may be prolonged. Re-biopsy for the purpose of detailed pathological and/or molecular characterisation must be considered to exclude second malignancy such as high-grade gliomas, a phenomenon increasingly reported [6,13,19,73,74,75,76,77,78,79]. Similarly, re-biopsy can also be helpful to exclude radiation necrosis [80]. Furthermore, molecular characterisation may help prognostication, with for example the identification of acquired driver events such as *TP53* mutations and *MYC* family oncogene amplification [1]. Looking towards the future, re-biopsy of rMB and identification of specific therapeutic targets may guide targeted treatment.

Finally, the indication for diversion of CSF should be established within a palliative context. The benefit of possible prolongation of survival, influence on mode of death, as well as the clinical condition of the patient must be carefully considered. Endoscopic third ventriculostomy (ETV) may be utilised to treat non-communicating hydrocephalus and re-establish flow from internal to external CSF spaces. This procedure could be combined with re-biopsy for possible intra- or paraventricular lesions. CSF diverting shunt systems may be used in communicating hydrocephalus in, for example, disseminated meningeal relapses. However, clinical deterioration due to tumour might be difficult to distinguish from shunt malfunction and shunt insertion may also give an alternative route for disease dissemination [81,82,83].

In summary, surgery should be considered to resect an isolated nodular lesion as this treatment may prolong survival, re-biopsy for the purpose of detailed pathological and molecular characterisation including exclusion of second malignancy, and palliative diversion of CSF to enhance quality of life. Importantly, re-biopsy for the purpose of molecular characterisation and exclusion of second malignancy should always be considered in experienced centres, wherever technically feasible.

### 5.2. Re-Irradiation

Re-irradiation offers the potential to decelerate tumour progression, reduce symptoms, and in some cases, provide long-term disease control [72,84]. Re-irradiation of the CNS is associated with the risk of cumulative toxicity, including brain necrosis. Furthermore, in children, re-irradiation is particularly challenging; pre-treatment burden such as systemic chemotherapy is higher when compared to adults and will contribute to the risk of cumulative toxicity. At present, few studies have investigated the factors determining tolerance of the CNS to re-irradiation in the paediatric population [85]. Currently, the Paediatric Normal Tissue Effects in the Clinic initiative (PENTEC) is dedicated to reviewing evidence and providing guidance on re-irradiation of the CNS and radiation-induced late effects in childhood cancer survivors [86]. In addition, the development of more precise irradiation techniques alongside smaller target volumes may allow safer re-irradiation while minimising the dose to surrounding critical structures [87]. There is now a growing number of studies reporting feasibility and low rates of second malignancies and radionecrosis after re-irradiation for rMB [72,84,88,89,90,91,92,93,94]. However, while both focal and CSI re-irradiation are increasingly considered as part of salvage therapy for rMB, only a minority of studies offer clear evidence for improved survival, which is highly dependent on principal molecular group and the re-irradiated site [72,84,88,93].

#### 5.2.1. Re-Irradiation Doses and Volume

There are currently no guidelines defining the concepts for re-irradiation, particularly in terms of dose, fractionation, modality, or techniques. Therefore, individualised strategies have to be established. Of particular importance when considering re-irradiation are the size and site of rMB, previous therapies, dose of initial CSI, time interval from initial CSI, neurological and general performance status, and proximity to critical organs at risk. As a rule, it is feasible to accept up to a total cumulative dose of 100 Gy to limited areas of the brain for conventional radiation techniques and up to 135 Gy with high-precision techniques such as stereotactic radiotherapy (SRT) and radiosurgery (SRS) [93,94,95,96]. Typically, a time interval of 6 months between first and second radiotherapy is acceptable and tolerated [72,97].

Given the reduction in initial CSI dose for low- and standard-risk MB (18–24 Gy), re-irradiation with second CSI may also be feasible albeit with unclear or mixed reports of additional benefit when compared to delivery of focal radiotherapy at relapse [72,94]. Most recently, Baroni et al. reported on 12 patients in receipt of second CSI. Re-irradiation was well tolerated; however, in assessable patients, neurocognitive deficit (mild-moderate intellectual disability) was frequently observed [72]. Doses at second CSI are similar to strategies at diagnosis; focal re-irradiation doses of up to 54 Gy and CSI doses of 20–36 Gy delivered as 1.6–1.8 Gy per fraction [72,84,94,98].

#### 5.2.2. Techniques

Modern, highly conformal radiation techniques such as SRT, SRS, PBT and intensity-modulated radiation therapy (IMRT) techniques can all be considered for re-irradiation.

#### 5.2.3. Stereotactic Radiotherapy/Radiosurgery

SRT and SRS allows high-dose treatment by intersecting multiple radiation beams in a precise three-dimensional target area. Both SRS and SRT have been described as effective, well-tolerated treatments for rMB achieving good disease control and prolonged progression-free survival in isolated solitary lesions or local relapses [85,89,99,100,101,102,103].

#### 5.2.4. Intensity-Modulated Radiation Therapy (IMRT)

IMRT is a highly conformal technique using photon beams. Based on computerised inverse planning, the intensity of the beam is modulated during treatment delivery which allows improved adaptation of the prescribed radiation dose to the target volume. However, due to the use of multiple fields, IMRT exposes larger amounts of normal tissue to low and medium doses of radiation, increasing the theoretical risk of second malignancies [104,105]. Importantly, however, IMRT can be applied to complex-shaped target volumes that are in close proximity to radiosensitive normal structures [106,107]. IMRT is a feasible and typically well-tolerated technique for re-irradiation of relapsed childhood brain tumours including MB [85,108,109,110].

#### 5.2.5. Proton Beam Therapy

**Figure 4:**
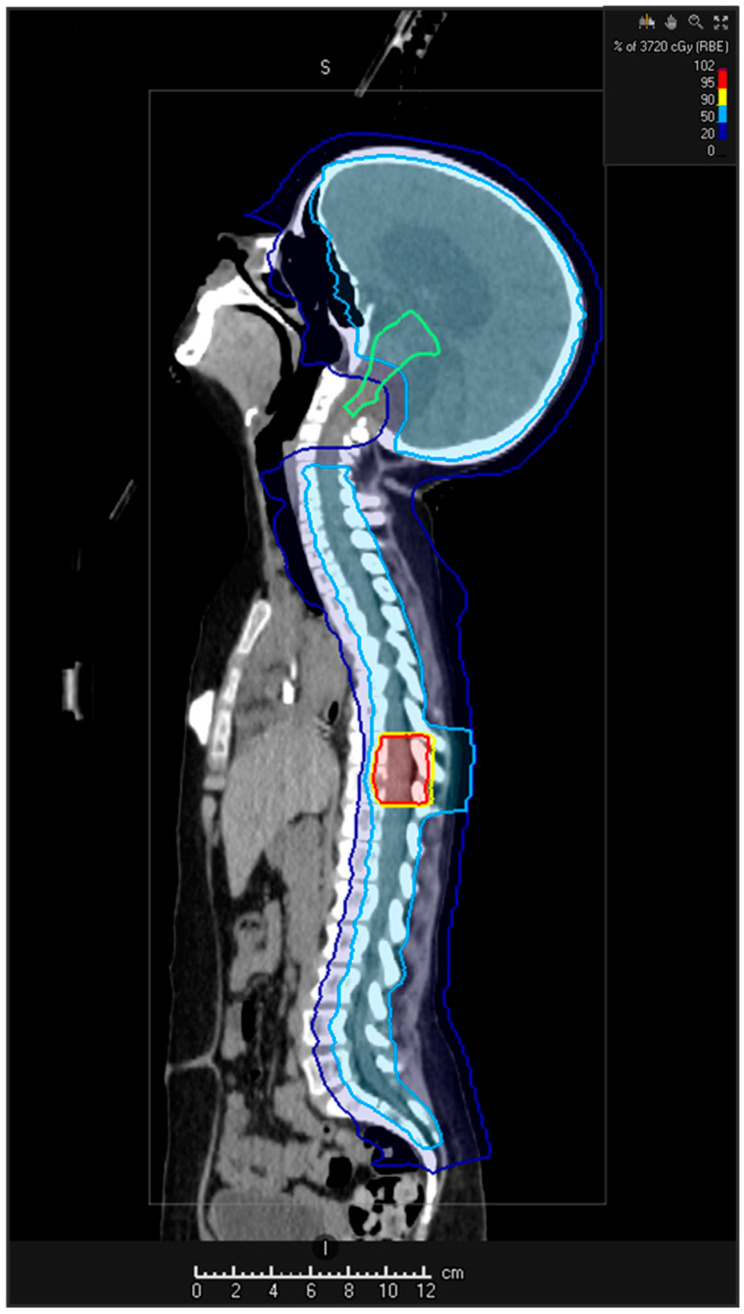
Re-irradiation for a rMB with a metastatic relapse. Second CSI (18 Gy) delivered with spinal boost (37.2 Gy). Integrated sparing of craniocervical junction and lower brainstem (green line) to avoid cumulative, intolerable doses. Initial CSI comprised 23.4 Gy with posterior fossa boost (54 Gy). Coloured areas reflect the CSI and boost volumes with respective doses displayed in the colour bar (top right panel).

The attractive dose distribution of protons is based on the energy loss of protons when interacting with tissue. This results in a steep energy deposition (Bragg Peak) when the beam ends allowing focused dose delivery and avoiding dose burden to the surrounding normal tissue [111,112]. PBT has become increasingly important in the treatment of childhood brain tumours and is a promising method for re-irradiation [113,114]. After introducing pencil beam scanning, intensity modulation with proton beam therapy can be applied offering highly individualised dose planning taking into account dose burden from previous radiotherapy (Figure 4). However, studies on re-irradiation with protons are sparse, predominantly focusing on recurrent paediatric ependymomas [115,116,117]. Early results of re-irradiation with protons in relapsed brain tumours displayed good feasibility [118]. Resection status before re-irradiation and a time interval ≥ 36 months between first and second irradiation were significantly associated with superior overall and progression-free survival.

In summary, there are a small number of studies demonstrating the safety of re-irradiation for rMB, with a minority of these studies offering evidence for improved survival, albeit highly dependent on molecular group and disease site. As more evidence emerges and initiatives such as PENTEC provide consensus guidance, the role of re-irradiation at relapse will become clearer. At present, however, delivery of re-irradiation for patients with rMB should be considered on an individual basis and in the context of other treatment options, prognostic factors, and patient/family wishes.

### 5.3. Chemotherapy

Given the dire prognosis of rMB following upfront multi-modal standard therapy, the aim of chemotherapy either as the sole treatment at relapse or combined with local therapy (neurosurgery and/or re-irradiation) is typically to prolong life with minimal side-effects/hospital admissions. The selection of chemotherapy regimen is influenced by numerous factors: previous treatment and associated toxicities, other severe co-morbidities, tumour burden and dissemination, anticipated disease course according to understood prognostic factors, and patient/family choice.

#### 5.3.1. High-Dose Chemotherapy

High-dose chemotherapy, defined as administration of intensive chemotherapy with autologous stem-cell support has been utilised in rMB for over two decades. Early reports in high-risk/relapsed brain tumours and follow-on studies in rMB demonstrated promising results with event-free survival rates as high as 30%. Regimens included busulphan, carboplatin, carmustine, cyclophosphamide, etoposide, melphalan and thioptepa. Toxicities were substantial and included hepatotoxicity, nephrotoxicity, CNS toxicity and treatment-related mortality [119,120,121,122,123]. Subsequent small, typically single-institute studies continued to evaluate treatment of rMB and other relapsed brain tumours with a variety of high-dose chemotherapy regimens which included the same agents as earlier studies alongside newer agents such as topotecan. Reported event-free survival varied greatly (0–56%). Furthermore, most studies again highlighted significant toxicities and the majority concluded that high-dose chemotherapy in rMB did not improve prognosis [91,124,125,126,127,128,129,130,131,132,133,134,135,136,137,138].

Two prospective national studies evaluated the role of high-dose chemotherapy for the treatment of rMB. The HITREZ 97 trial recruited 110 patients aged 0–30 years with relapsed or refractory MB, supratentorial primitive neuroectodermal tumour (PNET) or pineoblastoma, between 1997 and 2003. Treatment was non-randomised and comprised two regimens: oral chemotherapy (trofosfamide and etoposide) or intensive chemotherapy. In total, 72 patients (63 with relapsed/refractory (r/r) MB) received intensive chemotherapy which comprised a continuous infusion of carboplatin and etoposide followed by a disease response assessment after 2 cycles. A further 2 cycles were administered to patients with partial/complete responses and, subject to another response assessment, patients proceeded to high-dose chemotherapy (thiotepa-carboplatin-etoposide). Patients with metastatic disease also received intraventricular methotrexate. Twenty-seven of the 72 patients proceeded to high-dose chemotherapy. The median event-free survival (EFS) of these 27 patients was 8.4 months with 2, 3 and 5 year EFS of 20%, 10% and 0.1%, respectively. The median overall survival (OS) was 20.2 months with 2, 3 and 5 year OS of 35%, 20% and 17%, respectively. Toxicity was severe with an 8% treatment-related mortality rate and grade III–IV mucosal, haematological, infectious, and ototoxicity frequently observed. Ultimately, only 2 of the 72 patients (2.7%) selected for high-dose chemotherapy were reported to be in continuous remission [8].

The UKCCSG Relapsed PNET study (study number: CNS 2000 01) recruited 40 patients with rMB and 5 with relapsed PNET aged less than 21 years, between 2000 and 2007. Forty-four patients had received prior CSI (98%). High-dose chemotherapy comprised two sequential single-agent therapies (thiotepa followed by carboplatin) and was designed to minimise toxicities. All rMB patients (*n* = 40) initially received cytoreductive therapy (cyclophosphamide), 10/40 (25%) patients proceeded to thiotepa only, 12/40 (30%) patients received both thiotepa and carboplatin. Toxicities included hyperpigmentation, ototoxicity and fatal respiratory failure. Only 3/40 (8%) patients were alive at the time of reporting, one of whom had suffered a second relapse and was therefore unlikely to be cured long term [5].

#### 5.3.2. Conventional Chemotherapy

Multiple regimens of conventional chemotherapy have been utilised for the treatment of rMB [139,140,141]. Here, we summarise the most frequently reported/recommended regimens in the current era and their supporting evidence.

#### 5.3.3. Temozolomide, Irinotecan and Bevacizumab

Several case series have reported the use of temozolomide, irinotecan and bevacizumab for rMB [142,143,144,145]. Most recently, this regimen was one arm of the randomised phase II COG study, ACNS0821 (NCT01217437) designed to compare temozolomide and irinotecan with/without bevacizumab. One hundred and eight patients were recruited (3 were subsequently ineligible), aged less than 21 years old, between 2010 and 2015. Eighty-five of the 105 assessable patients (81%) had r/rMB, the majority of whom received upfront CSI. Fifty-three patients (r/rMB = 44) were randomised to receive temozolomide and irinotecan (two-drug regimen) and 52 (r/rMB = 41) to temozolomide, irinotecan and bevacizumab (three-drug regimen). Toxicities were comparable between the regimens with gastrointestinal disturbances, electrolyte imbalances, liver function abnormalities and myelosuppression most frequently observed. Importantly, there was only one patient in each treatment arm who suffered an intracranial bleed. In the two-drug regimen, two patients died of sepsis; in the three-drug regimen, 3 patients were removed from the study due to unacceptable toxicities. Only 23 patients completed 12 cycles of chemotherapy, 21% in the two-drug regimen and 23% in the three-drug regimen. For patients with r/rMB, median EFS and OS were 5 and 11 months, respectively, in the two-drug regimen,; 10 and 19 months, respectively, in the three-drug regimen. Based on the initial study aims, the authors concluded that the three-drug regimen significantly reduced the risk of death in r/rMB (one-sided *p* = 0.024) and warranted further investigation [146].

#### 5.3.4. Temozolomide and Irinotecan (TEMIRI)

Temozolomide with irinotecan is a frequent salvage strategy in relapsed childhood tumours [147,148]. One multi-institutional ITCC/SIOPE single-arm phase II trial has been undertaken between 2007 and 2010 in rMB (NCT NCT00404495). In total, 66 patients aged 6 months to 18 years with rMB were recruited, 59 of whom (89%) had received upfront CSI. Fifty-one patients had suffered 1 relapse, 15 patients had had 2 or more relapses. Overall, 48 patients had evaluable disease for the primary endpoint: objective tumour response during the first 4 cycles of treatment. Eighteen patients were not evaluable due to incomplete assessments or poor imaging quality. Secondary endpoints included best overall tumour response, duration of tumour response, time to tumour progression, time to treatment failure, and OS. The median number of cycles started was 6, with most patients terminating treatment due to disease progression. Frequent treatment-associated toxicities were gastrointestinal upset, haematological toxicities, and electrolyte disturbances. Objective tumour response rate after 4 cycles was 32.6% and did not meet the desired target of 40%. Median EFS and OS were 4.3 and 16.7 months, respectively [149].

#### 5.3.5. Temozolomide and Topotecan (TOTEM)

The combination of temozolomide and topotecan (TOTEM) has also been utilised across a range of relapsed childhood tumours [150,151]. Most recently, an international multi-centre, non-randomised, phase II study conducted between 2009 and 2013 investigated this regimen’s efficacy in childhood brain and extracranial solid tumours (NCT00918320). Importantly, this study included an expansion cohort of 20 patients with r/rMB following promising disease-specific results in the initial 9 patients. The primary endpoint was objective response rate after 2 cycles of chemotherapy. Secondary endpoints were best tumour response, duration of overall response, EFS and OS. The most frequently observed grade III and IV toxicities were haematological, febrile neutropenia, and liver function abnormalities. In total, 29 patients aged 6 months to 20 years with r/rMB were included in this study, the majority of whom (17/29, 58%) had received upfront CSI. The objective response rate of the r/rMB cohort was 28%. The median EFS and OS were 2.8 and 12.2 months, respectively [7].

#### 5.3.6. Temozolomide

Temozolomide is a frequently utilised single-agent option for the treatment of rMB. Typically, it has been administered over 5 days in 28-day cycles although alternative, metronomic style regimens over 42 days have also been explored. [152,153,154]. Early-phase trials in relapsed brain tumours demonstrated responses in rMB. One study, comprising 25 evaluable patients with rMB/PNET, reported an overall response rate of 16% [155]. The most recent phase II trial exploring temozolomide for the treatment of rMB/PNET recruited 39/42 (93%) patients with rMB aged 2–21 years. The most frequently observed grade III and IV toxicities were haematological; thrombocytopenia, neutropenia, and anaemia. Non-haematological toxicities were minimal such as nausea and vomiting. For the entire evaluable cohort in this study (*n* = 40) overall response rate was 42.5%, EFS was 30% and 7.5% at 6 and 12 months; OS 42.5% and 17.5% at 6 and 12 months, respectively [156].

#### 5.3.7. Metronomic Chemotherapy

Metronomic chemotherapy, defined as the chronic administration of chemotherapeutic agents without prolonged drug-free breaks, at low, minimally toxic doses, is believed to work by inhibiting tumour growth through antiangiogenic mechanisms, promoting apoptosis and immune-surveillance [157,158,159]. The most frequently observed toxicity is myelosuppression but it is important to note that secondary leukaemias have been reported following prolonged exposure to metronomic therapy [157,159]. Both single-agent and combination metronomic therapy have been utilised for rMB.

#### 5.3.8. Etoposide

Low-dose oral etoposide has been administered for relapsed brain tumours for over 20 years, with frequent stabilisation of disease, occasional responses and minimal toxicity reported [160,161,162]. While there have been no recent trials examining the efficacy of oral etoposide in rMB alone, its use is still commonplace [163]. However, prolonged oral etoposide therapy with high cumulative doses poses the risk of secondary leukaemia [164,165].

#### 5.3.9. Metronomic and Targeted Antiangiogenesis Therapy for Children with Recurrent/Progressive Medulloblastoma (MEMMAT)

Initial studies examining the utility of bevacizumab, thalidomide, celecoxib, fenofibrate, etoposide alternating with cyclophosphamide, and intrathecal therapy (alternating liposomal cytarabine and etoposide) in relapsed brain tumours reported encouraging results in patients with rMB (*n* = 7). Toxicities included neutropenia, infection, peripheral neuropathy secondary to thalidomide, hypothyroidism, proteinuria and haematuria. Papilloedema and raised intracranial pressure were noted following intraventricular liposomal cytarabine in 2 patients. As a result of these early findings, a phase II study, MEMMAT (NCT01356290), was opened [159]. An interim report of the first 29 patients recruited to MEMMAT between 2006 and 2016 has recently been presented, albeit the trial is still recruiting [166]. All 29 patients had rMB (19 first relapse and 10 multiple relapses) with a median age of 10 years (range 1–27 years). Currently, 8/29 (27%) patients are alive with 6/8 in continuous complete remission. Treatment was reported to be well tolerated, although five patients died of other causes such as secondary leukaemia and sepsis. EFS was 33% and 28% at 5 and 10 years, OS was 44% and 39% at 5 and 10 years, respectively [166]. We await the results of the MEMMAT trial which is due to complete in 2023.

#### 5.3.10. Modified MEMMAT

It is not uncommon for individual clinicians to adapt the MEMMAT regimen to meet the needs of their patient, minimise toxicity or conform to regulatory requirements where some agents are not readily available. A common adaptation includes the administration of alternating 3 weekly cycles of etoposide and cyclophosphamide/trofosfamide alone or one weekly cycles of temozolomide and topotecan (unpublished data HIT-REZ-2005 study and HIT-REZ-Registry cohort). Pre-clinical and clinical evidence for these approaches are not available but anecdotally they are typically well tolerated and may prolong survival.

#### 5.3.11. Combined Oral Metronomic Biodifferentiating Antiangiogenic Treatment (COMBAT)

A variety of COMBAT regimens have been reported, predominantly for the treatment of paediatric solid tumours. Agents utilised include low-dose temozolomide, etoposide, 13-cisretinoic acid, celecoxib, vitamin D, fenofibrate, and sodium valproate [167,168,169]. A retrospective study undertaken between 2011 and 2016, on patients with high-risk or rMB reported on 39 children, 20/39 (51%) of whom had rMB and received low-dose temozolomide, etoposide, sodium valproate and 13-cisretinoic acid in 12-weekly cycles. Treatment was generally well tolerated, albeit one child developed secondary leukaemia. Two-year EFS and OS for patients with rMB were 25.8% and 67.4%, respectively [169]. Unlike MEMMAT, however, the COMBAT regimen has not been taken forward into a clinical trial for rMB.

#### 5.3.12. Temozolomide and Etoposide

Other agents that have been explored as metronomic therapy for r/rMB include alternating temozolomide and etoposide. An initial phase I study undertaken between 2005 and 2008 identified the maximum tolerated dose for the combination of temozolomide and etoposide for children aged between 3 and 18 years with r/rMB [170]. Fourteen patients were recruited, 9 of whom had rMB and 13/14 (93%) had received upfront CSI. Treatment was generally well tolerated with thrombocytopenia, neutropenia and anaemia being the most common adverse events. As a phase I study, response was not an endpoint and was not formally reported. Furthermore, no phase II study has been undertaken [170].

In summary, there are a wide range of high-dose, conventional and metronomic chemotherapy regimens that have been utilised and/or formally trialled in rMB. High-dose chemotherapy regimens do not confer a survival advantage, are highly toxic and are therefore not recommended for use in pre-irradiated (CSI) patients who relapse [171]. Of the conventional chemotherapy regimens, temozolomide, TOTEM, TEMRI, and temozolomide/irinotecan and bevacizumab, are the most frequently utilised regimens, typically well tolerated, albeit with an increasing number of potential side-effects, respectively. Phase II trial data are available for all these regimens and report comparable prolongation of survival [7,146,149,156]. Finally, metronomic therapy, such as MEMMAT and COMBAT regimens, are well tolerated, with early reports of benefit. However, neither regimen has complete trial data available, with MEMMAT being the only metronomic trial recruiting rMB patients.

### 5.4. Targeted Therapies

#### 5.4.1. MB_SHH_: Smoothened Inhibitors

The SHH pathway is an appealing target in MB_SHH_ and inhibitors of Smoothened (SMOi) have been developed and approved for use in basal cell carcinoma [172]. Responses to SMOi have, as expected, only been reported in MB_SHH_ with SHH pathway activation upstream of *SMO* [173]. In the phase I clinical trial of vismodegib, antitumor activity was seen in one third of evaluable patients with MB_SHH_ [174]. In the phase I study of oral sonidegib, significant activity in both adult and paediatric MB_SHH_ patients was reported, with no responses observed in tumours without activation of the SHH pathway [175]. More recently, a retrospective study by Pereira et al. reported responses in all rMB patients with somatic or germline *PTCH1* mutations (6/8, 75%), including 2/8 (25%) with prolonged responses. Importantly, several of these studies reported growth plate fusion as a major side-effect of SMOi, limiting their upfront use to patients who are skeletally mature [175,176,177]. Furthermore, despite very good or complete responses, resistance to SMOi frequently occurred while on treatment [176].

Resistance mechanism involving mutations within *SMO* are well recognised in basal cell carcinoma and have importantly been described in both pre-clinical and early-phase MB clinical trials [36,172,178,179,180]. Furthermore, a recent single-cell (sc) RNAseq study demonstrated that at the outset of treatment with vismodegib, murine MB_SHH_ demonstrated serval cell populations no longer depended on the initiating oncogenic mutation and, in the absence of a prolonged period of selective inhibition with vismodegib, conferred resistance to SHHi [181].

In summary, the most effective and least toxic way to implement SMOi for the treatment of MB_SHH_ remains to be defined; several studies have now reported growth plate fusion as a major side-effect of SMOi [175,176,177]. Pereira et al. suggests SMOi for the treatment of rMB_SHH_ should be time limited, a bridge to local therapy (neurosurgical resection/radiotherapy) and delivered in combination with other therapies to prevent selection of one or several resistant clones [176].

#### 5.4.2. MB_Group3_- and MB_Group4_-Targeted Therapies

The underlying molecular drivers for MB_Group3_ and MB_Group4_ remain to be fully characterised and therefore no specific targeted treatments are currently available. Indirectly targeting *MYC* continues to be explored in MB_Group3_ with therapies such as bromodomain inhibitors [182,183,184,185,186]. For MB_Group4_ there may be a role for epigenetic-based therapies such as demethylating agents (decitabine and azacitidine) and histone deacetylase inhibitors (vorinostat and panobinostat) [182,187,188]. New insights into rMB_Group4_ have also identified emergent and potentially targetable events such as *CDK6* amplification [6]. Undoubtedly, however, new strategies to identify molecular drivers are required across all the MB molecular groups and include initiatives such as SMPaeds, INFORM and MAPPYACTS [14,15,16,17].

### 5.5. Immunotherapy

#### 5.5.1. Checkpoint Inhibitors

Immune checkpoints are molecules that are activated to initiate an immune response; their role is to maintain immune homeostasis and to prevent auto immunity. Cancer cells can use these checkpoints, such as PD-L1 and cytotoxic T-lymphocyte-associated protein 4 (CTLA4), to avoid T-cell-initiated apoptosis. The activity of checkpoint inhibitors such as nivolumab therefore depends on the expression of PD-L1 in tumour cells.

In general, paediatric MB is a ‘cold’ tumour, with a low-level of PD-L1 expression and a low mutational burden [55,189,190,191,192]. The higher the level of tumour mutations, the larger the chance that neoantigens will be recognised by T cells, making checkpoint blockade more effective. Furthermore, the immune micro-environment of MB is non-inflammatory and subsequently does not recruit immune cells [190,193]. However, a small number of in vitro studies have shown distinct micro-environmental phenotypes according to molecular group and consequently differing responses to immune checkpoint blockade [190,194,195,196]. For example, the highest PD-L1 expression is found in MB_SHH_. Furthermore, in murine MB_SHH_ models there is a higher percentage of infiltrating immune cells compared with MB_Group3_ models [194,196]. As such, several checkpoint inhibitors including nivolumab, prembrolizumab and durvalumab are currently under investigation in variety of relapsed and refractory brain tumours (NCT03173950, NCT02359565 and NCT02793466, respectively).

#### 5.5.2. Oncolytic Viruses

Oncolytic viruses can replicate in cancer cells but not in normal cells. Oncolytic viruses induce an immune response against themselves and the infected tumour cells which both kills the tumour cell and facilitates immunological memory [197]. Most pre-clinical studies are performed on PDX models in immune-deficient hosts with intratumoral injection of viruses. However, because immunity against viruses in patients is prevalent, results from immunodeficient mouse models may not translate to human disease [198].

Several viruses, both unmodified (e.g., reovirus) and genetically engineered for tumour selectivity and enhanced immune stimulation (e.g., herpesvirus, poliovirus, adenovirus, and measles virus) are being investigated in paediatric brain tumours [198]. For example, expression of the measles and polio virus receptors (CD46 and CD155) have been investigated in MB cell lines and patient samples in vitro [199,200]. CD155 was expressed on MB cell lines and an in vitro study demonstrated that the polio: rhinovirus recombinant (PVSRIPO) was capable of inhibiting cell proliferation and killing MB_Group3_ [199]. Moreover, infection of MB cell lines with measles virus showed significant killing, and treatment of a MB patient-derived xenograft (MB-PDX) with activated measles virus led to an increase in survival compared with the with inactivated measles virus [200]. Furthermore, in a metastatic MB-PDX model, intraventricular administration of measles virus resulted in tumour stabilisation and shrinkage, significantly prolonging OS of the treated animals compared with those treated with an inactivated virus [199,201,202].

In human trials, a recent phase I study with oncolytic HSV-1 G207 immunovirotherapy for paediatric high-grade gliomas showed an acceptable side-effect profile with evidence of responses [203]. Several other viruses are under phase I testing including PVSRIPO, Delta-24-RGD adenovirus, modified measles virus, and herpes simplex virus G207 (NCT03043391, NCT04758533, NCT02962167, and NCT03911388, respectively) [198,204,205].

#### 5.5.3. Chimeric Antigen Receptor Therapy (CAR T-Cell Therapy)

CAR T-cells are a form of adoptive cell therapy. Ideally, the CAR-target-specific antigens are highly and homogeneously expressed across tumour cells, and absent or display low expression on normal tissue [206,207,208]. These CARs allow T cells to recognise and attach to a specific protein or antigen on the tumour and subsequently kill the tumour cell. In paediatric brain tumours, current antigenic targets include B7-H3, GD2, IL-13Rα2, EphA2, and HER2, with an emerging body of pre-clinical evidence in PDX suggesting potential therapeutic benefits from CAR T-cell therapy [206,209,210]. One study has demonstrated the use of locoregional delivery of EPHA2 CAR T-cells as a highly effective therapy in MB_Group3_ PDX [211].

CAR T-cell therapy for brain tumours has associated toxicity including cytokine release syndrome, which can lead to oedema and associated hydrocephalus [212]. However, several early-phase clinical trials are currently recruiting, utilising different antigens as targets such as B7-H3 and HER2, to assess the safety and efficacy of CAR T-cell therapy in paediatric brain tumours (NCT04185038, NCT02442297 and NCT03500991). An early report by Vitanza et al. has demonstrated the feasibility of generation and repeated locoregional delivery of HER2-specific CAR T-cells in three patients (two ependymoma and one anaplastic astrocytoma) with no dose limiting toxicities and importantly evidence of local CNS immune activation (NCT03500991) [213].

In summary, immunotherapy is under development in many different forms, and has shown great potential in pre-clinical and early-phase clinical trials [204,205,206]. However, despite the remarkable clinical efficacies observed in a number of malignancies, the use of immunotherapy alone may not be sufficiently active for many patients. Therefore, it is likely that the real potential for most patients, including those with r/rMB, lies in combining different immunotherapies with other treatments such as radiotherapy and chemotherapy to target tumour heterogeneity, overcome immunosuppression, and improve immune infiltration [211,214,215].

### 5.6. Intrathecal Therapies

Intrathecal chemotherapy has been used since the 1980s for the treatment of brain tumours, including rMB. The main aim of this approach in rMB is to treat or prevent spread of tumour cells in the ventricular/subarachnoid space and LMD [216]. Intrathecal therapy can achieve high drug concentrations in the CSF, but it is usually ineffective in treating bulky (nodular) LMD or parenchymal disease due to poor drug penetration, [217]. Furthermore, drug distribution via the CSF can vary considerably depending on the delivery route (intraventricular versus intralumbar route) and pattern of disease relapse. Following intraventricular delivery, volume of drug distribution is greater and importantly independent of body position, in contrast to the intralumbar route [218,219,220]. LMD itself may affect drug distribution in the CSF as well as drug absorption and elimination from the CSF. This may either raise local drug exposure, conveying a higher risk of arachnoiditis, encephalopathy and myelopathy, or may have the opposite effect of preventing sufficient drug distribution. Additionally, patients with a ventriculo-peritoneal (VP) or ventriculo-atrial (VA) shunt have unpredictable CSF dynamics and consequent drug distribution. Finally, drug elimination can be delayed in patients with high CSF protein levels and in patients with increased intracranial pressure [216,221].

#### 5.6.1. Intrathecal Chemotherapy

The most commonly used intrathecal chemotherapies for the treatment of LMD in rMB are methotrexate, cytarabine (transiently used in its liposomal formula until production was discontinued), and etoposide [159,222,223,224]. There is no established standard intrathecal chemotherapy and a paucity of controlled trials both in newly diagnosed and rMB. However, over the last four decades, several treatment regimens of intrathecal therapy have been investigated [216,225].

It has been demonstrated that for methotrexate, cytarabine, topotecan and etoposide, repetitive intraventricular delivery of small doses is feasible, preventing high peak levels and providing prolonged cytotoxic drug levels. This regimen leads to high drug exposure with documented responses in the form of tumour cell clearance in the CSF and/or LMD stabilisation [223,226,227,228,229]. Long-term exposure can also be achieved by continuous ventriculolumbar CSF perfusion, which is not routinely used due to the complexity of this approach [230]. Each intrathecal agent, especially in LMD, conveys a risk of acute or delayed neurotoxicity such as arachnoiditis, radiculitis, encephalopathy, and myelopathy. Arachnoiditis in particular, was observed with liposomal cytarabine prior to its withdrawal, necessitating the use of dexamethasone prophylaxis [159]. In light of these toxicities, concurrent radiotherapy and intrathecal chemotherapy must be avoided. Methotrexate in particular conveys a higher risk of encephalopathy and must not be delivered after radiotherapy [231,232,233,234,235]. Finally, a small number of other agents (e.g., diaziquone, 6-mercaptopurine, mafosfamide, and busulfan) have been investigated in clinical trials or experimental studies; none of which have become standard of care [216].

#### 5.6.2. Intrathecal Immunotherapy

There is an emerging body of early-phase trials examining the use of intrathecal immunotherapy in childhood brain tumours. A phase II study evaluating intrathecal radioimmunotherapy with ^131^I-labelled monoclonal antibody 3F8, which targets the cell-surface disialoganglioside GD2, has demonstrated feasibility, manageable toxicity and clinical utility in high-risk and rMB [236]. In an ongoing phase I/II trial, the safety and efficacy of intracerebroventricular radioimmunotherapy using 177Lu-DTPA-Omburtamab which targets B7-H3, an immune checkpoint molecule that is widely expressed in tumour cells, will be investigated in r/rMB (NCT04167618). Other recent studies in recurrent and refractory CNS tumours, including r/rMB, will evaluate locoregional CAR T-cell therapy specific for IL13Ralpha2, EGFR806, HER2 and B7-H3, respectively. In these studies, CAR T-cells will be delivered directly into the resection cavity or the ventricular system (NCT04661384, NCT03638167, NCT03500991, and NCT04185038).

In summary, intrathecal therapy is not standard of care for r/rMB due to a lack of clinical trials. However, there is a body of evidence demonstrating efficacy in eliminating tumour cells from the CSF potentially leading to prolonged disease stabilisation. Intrathecal therapy should be considered on an individual basis in patients with disseminated LMD. This is particularly true if there is limited bone marrow reserve restricting the use of systemic and more intensive chemotherapy, or if there is not an option for localised therapy such as neurosurgery or re-irradiation. The intraventricular route via an Ommaya or Rickham Reservoir is the preferred method of delivery; this enables fractionated low doses, improving drug exposure and preventing potentially neurotoxic peak levels. However, unpredictable CSF drug concentrations in disturbed CSF dynamics require experienced teams to undertake these regimens with close clinical, cytological and drug monitoring of these patients. This level of intervention and hospital time alongside the potential risk of neurotoxicity may not be appropriate or acceptable to all patients. Ongoing and future trials will investigate the role of intrathecal chemotherapy and targeted therapy in form of radioimmmunotherapy or specific CAR T-cell therapy in rMB.

## 6. Supportive Care and Follow-Up Investigations

Supportive care for all patients, regardless of whether they are receiving active treatment, focuses on enhancing quality of life and symptom management. Shared decision making, establishing goals of care, and patient/family values are key to achieving these objectives. Furthermore, as our understanding of the molecular landscape of rMB expands and additional aberrations associated with disease behaviour are identified, this information can be utilised to guide conversations (see Prognostic Factors). Patients with rMB can have varied and complex symptoms depending on the disease location with local relapses leading to cerebellar signs (see Signs and Symptoms), brainstem involvement disrupting cranial nerves resulting in speech and swallowing difficulties, supratentorial disease being more likely to result in seizures, and spinal disease posing the risk of paralysis. A multi-disciplinary team approach is therefore best practice to provide for the holistic needs of the child and their family [237].

Follow-up investigations to assess disease response must be tailored to the individual and consider patient and family wishes. Those patients on an early-phase trial should, wherever possible, follow the trial protocol and stipulations. It is therefore important to counsel patients and their family for these likely additional investigations and time in hospital prior to consenting for the early-phase trial. Similarly, it is best practice to assess disease response while receiving more intensive treatment outside of a trial setting, such as conventional chemotherapy (see Chemotherapy). Conventional chemotherapy regimens (e.g., temozolomide, irinotecan and bevacizumab, TEMIRI, and TOTEM) are likely to cause toxicity and therefore confirming benefit in the form of stable disease or partial/complete response is important information to balance against continuation of treatment. However, the nature (MRI and serial lumbar punctures; see Staging) and timings of reassessment will vary depending on the treatment itself, degree of toxicity, and patient/family wishes. Furthermore, age of the patient will determine the need for general anaesthetic when undertaking, for example, an MRI and may also influence follow-up.

In summary, patients with rMB can have complex symptoms requiring a holistic multi-disciplinary team approach. Supportive care should focus on enhancing quality of life and symptom management, with shared decision making guided by goals of care, family and patient wishes, and relevant prognostic factors.

## 7. Future Developments

A greater understanding of treatment resistance and the biological mechanisms underpinning early therapy-refractory and relapse disease are urgently required across childhood cancers, including MB. Initiatives are emerging to undertake; relapsed tumour biopsies matched with early-phase clinical trials, liquid biopsies, and modelling strategies which more accurately recapitulate disease heterogeneity and subsequent clonal evolution.

### 7.1. Drug–Target Matched Clinical Trials

There are now multiple international studies (SMPaeds, INFORM, MAPPYACTS, eSMART, MATCH and PRISM) which seek to molecularly characterise high-risk and relapsed childhood tumours, identify a potential therapeutic target, and commence an appropriate targeted treatment [14,15,16,17]. Established molecular profiling strategies such as DNA methylation analyses, copy number profiling and next-generation sequencing necessitate appropriate and timely collection of tumour material. Importantly, DNA and RNA extracted from freshly frozen material performs best across all platforms and is a requirement for whole-genome sequencing approaches at present (Figure 3). Collection and handling of potentially limited tumour material should therefore be discussed between the paediatric oncology, neurosurgical and neuropathology teams prior to re-biopsy to best facilitate clinical requirements (see Molecular Pathology), explore the ability to submit material for extended molecular profiling, and set appropriate expectations for patients and their family.

In summary, through the advancement of drug–target matched clinical trials, there is the opportunity to molecularly characterise rMB. Collection, handling, and coordination of tumour material to facilitate both clinical and research needs should be discussed and planned prior to biopsy.

### 7.2. Liquid Biopsies

Sources for liquid biopsy, known as the liquid biome, include blood, urine and CSF. Liquid biopsies work on the principle that tumours shed detectable biological material into the liquid biome, with tumours proliferating at a high rate more likely to shed this material [238,239].

Biological materials including circulating tumour DNA (ctDNA), circulating tumour cells (CTC), microRNA (miRNA), and proteins/peptide fragments, have all been investigated within the liquid biome. The detection of ctDNA, fragments of DNA shed by the tumour cells, has been reported in childhood brain tumours such as diffuse intrinsic pontine gliomas (DIPG). Liquid biopsies of blood and CSF have successfully identified tumour-specific events, such as H3K27M mutations in DIPG, using droplet digital polymerase chain reaction (PCR) methods [238,240,241]. However, due to the blood–brain barrier, CSF serves as a better source than blood for obtaining ctDNA for most childhood brain tumours [242]. In first MB studies, both tumour-specific mutations and differentially methylated CpGs have been utilised to isolate ctDNA from CSF [243,244]. More recently, Liu et al. demonstrated clear utility of low-coverage whole-genome sequencing to detect copy-number variations (CNV) as a biomarker of MRD. For example, detection by ctDNA of MRD in patients with complete responses proceeded disease relapse on imaging by ≥3 months [242].

While ctDNA has the advantage of small sample requirement it does require the presence of a unique genomic/epigenomic marker for detection, such as tumour-specific CNV or mutations. Alternatively, CTC provide a wealth of potential genomic, transcriptomic and epigenomic information but require greater sample volumes and remain challenging to isolate due to their rarity. While they have not yet been studied widely in childhood brain tumours, CTC have been detected in the blood of adult glioblastoma patients, providing proof of concept for future studies [240]. Other possibilities include detection of miRNA, which regulates mRNA and has a role in disease biology for a variety of tumours including MB [245]. Studies are emerging for the detection of circulating miRNA in the blood of children with brain tumours such as astrocytomas. Finally, there is emerging research exploring profiling proteins in the CSF using mass spectrometry for the detection of tumour associated peptides and protein fragments [240].

In summary, liquid biopsies of CSF in childhood brain tumours could provide a route to safely monitor disease response, early diagnosis of disease progression/relapse, evaluate LMD, serial surveillance, and identify molecular targets. Challenges include disease rarity, sampling difficulties, paucity of matched control samples, and need for cost effective platforms. However, with continued technological advancements, optimisation of isolation methods and analytical tools, liquid biopsies have real potential to improve early detection and outcomes for patient with rMB [239,240,242].

### 7.3. Modelling Strategies

Creating in vitro and in vivo MB models representative of both intra- and intertumoral heterogeneity is a key area of pre-clinical development required to advance novel therapies for rMB. Patient-derived cell lines and organoids remain a challenge to develop in MB, albeit success is observed in other brain tumours such as glioblastoma [246,247]. Repositories of MB-PDX are however available both commercially and within the research setting [248,249,250]. Successful engraftment is not however guaranteed, and only approximately 40% of all MB engraft in vivo, with over representation of the more aggressive phenotypes/genotypes such as *MYC* amplified MB_Group3_ observed [250,251]. Several studies have successfully used MB-PDX as part of pre-clinical trials [211,249,251]. Other approaches include the use of established MB-PDX lines for in vitro drug screens. In one study, Rusert et al. demonstrated that it was the gene expression profiles of a panel of MB-PDX, rather than mutational aberrations, which best identified target-drug matches. These findings were subsequently validated in vitro utilising the panel of MB-PDX lines, albeit in vitro responses did not consistently match with target-drug predictions. This study also went on to show that drug prediction analyses can be undertaken on molecular profiles of patients’ tumours in conjunction with in vitro drug screening of tumour cells in real-time, adding a new dimension to the concept of precision medicine and individualised therapy [251].

In summary, developing in vitro and in vivo models which represent both intra- and intertumoral heterogeneity remains a challenge. Key areas of development include the utility of patient-derived models for drug screens/drug prediction analyses.

## 8. Conclusions

In conclusion, while rMB remains a significant area of unmet clinical need in paediatric oncology, current and future initiatives provide hope for new approaches to modify outcomes. Current state-of-the-art approaches to the diagnosis and management of rMB comprise comprehensive radiological, histological and molecular assessments, consideration of local therapy options (surgery and/or re-irradiation) and a selection of chemotherapy regimens which can be utilised on an individual patient basis. Importantly, early-phase trials should always be explored in the context of rMB and useful contemporary resources include kidscancertrials.ucsf.edu, itcc-consortium.org, and clinicaltrials.gov.

If we continue to intensify our national and international collaborative pre-clinical and clinical research through profiling strategies of rMB and, for example, liquid biopsies, our understanding of disease evolution and the molecular drivers of rMB will expand. Coupling these efforts with more representative modelling strategies and advancement of immuno- and targeted- therapies, we look towards a future where we either prevent relapse from occurring in the first instance or have curative options available for a decreasing number of patients who suffer from rMB.

## Figures and Tables

**Table 1:** Essential and optional brain Magnetic Resonance Imaging sequences. * 3D FLAIR can be used instead of 2D FLAIR but not if 2D sequences have been used for the same individual on previous occasions. ** The heavily weighted T_2_W sequence localised to a region of interest is useful in assessment of lesions (in particular poorly/non-enhancing) within the extra-axial space or along the parenchymal surface. SE, Spin Echo; TSE, Turbo Spin Echo; FSE, Fast Spin Echo; MP-RAGE, Magnetisation-Prepared Rapid Gradient-Echo; IR, Inversion Recovery; SPGR, Spoiled Gradient Recalled echo; TFE, Turbo Field Echo; FFE, Fast Field Echo; DWI, diffusion-weighted imaging; ADC, Apparent Diffusion Coefficient; EPI, Echo Planar Imaging; CISS, Constructive Interference in Steady State; bFFE, balanced Fast Field Echo; FIESTA, Fast Imaging Employing Steady-state Acquisition; DTI, Diffusion Tensor Imaging; NA, not applicable.

Essential Sequences: 1.5 Tesla Scanner			
Sequence	Technique	Parameters	Plane
T_1_W	2D SE, TSE/FSE	Slice thickness ≤4 mm	Axial (along AC-PC axis)
		Slice gap ≤1 mm (10% of slice thickness desirable)	
T_2_W	2D SE, TSE/FSE	Slice thickness ≤4 mm	Axial
		Slice gap ≤1 mm (10% of slice thickness desirable)	
T_2_ FLAIR	2D TSE/FSE	Slice thickness ≤4 mm	Axial or coronal
		Slice gap ≤1 mm (10% of slice thickness desirable)	
T_1_W + contrast	2D SE, TSE/FSE	Slice thickness ≤4 mm	Axial, coronal and sagittal
		Slice gap ≤1 mm (10% of slice thickness desirable)	
DWI with ADC	2D EPI	Slice thickness ≤4 mm	Axial
		Slice gap ≤1 mm (10% of slice thickness)	
		b = 0 and 1000. ADC maps reconstructed on-line	
**Essential Sequences: 3 Tesla Scanner**			
**Sequence**	**Technique**	**Parameters**	**Plane**
T_1_W	3D gradient echo (MP-RAGE/IR-SPGR/Fast SPGR/3D TFE/3D FFE)	Slice thickness ≤1 mm with no slice gap	Axial or sagittal
		Isotropic voxel resolution of 1 mm × 1 mm × 1 mm desirable	
T_2_W	2D SE, TSE/FSE	Slice thickness ≤4 mm	Axial
		Slice gap ≤1 mm (10% of slice thickness desirable)	
T_2_ FLAIR	2D TSE/FSE	Slice thickness ≤4 mm	Axial or coronal
		Slice gap ≤1 mm (10% of slice thickness desirable)	
T_1_W + contrast	2D SE, TSE/FSE	Slice thickness ≤4 mm	Axial
		Slice gap ≤1 mm (10% of slice thickness desirable)	
T_1_W + contrast	3D gradient echo (MP-RAGE/IR-SPGR/Fast SPGR/3D TFE/3D FFE)	Slice thickness ≤1 mm with no slice gap	Axial or sagittal, to match pre-contrast
		Isotropic voxel resolution of 1 mm × 1 mm × 1 mm desirable	
DWI with ADC	2D EPI	Slice thickness ≤4 mm	Axial
		Slice gap ≤1 mm (10% of slice thickness desirable)	
		b = 0 and 1000, ADC maps reconstructed on-line	
Resolution parameters: Field of view—230 mm (range 220–250 mm depending on head size). Matrix size—minimum 256 (512 is desirable for better resolution; 96–128 for EPI sequences).			
**Optional Sequences**			
**Sequence**	**Technique**	**Parameters**	**Plane**
T_1_W	3D gradient echo (on 1.5 T)/3D T1 TSE	-	Axial or sagittal
T_2_ FLAIR	3D gradient echo *	-	Axial or sagittal
Heavily weighted T_2_W	2D or 3D CISS/bFFE/FIESTA **	-	Axial or coronal or sagittal
Advanced MRI	DTI, perfusion and spectroscopy	-	NA

**Table 2:** Essential and optional spinal Magnetic Resonance Imaging sequences. * In primary tumours of the spinal cord, T and pre contrast T_1_W sequences are essential. ** The heavily weighted T sequence localised to a region of interest is useful in assessment of drop metastasis. SE, Spin Echo; TSE, Turbo Spin Echo; FSE, Fast Spin Echo; CISS, Constructive Interference in Steady State; bFFE, balanced Fast Field Echo; FIESTA, Fast Imaging Employing Steady-state Acquisition.

Essential Sequences			
Sequence	Technique	Parameters	Plane
T_1_W + contrast	2D SE/TSE	Slice thickness ≤3 mmSlice gap <0.5 mm	Sagittal whole spine (entire dural sac)
T_1_W + contrast	2D SE/TSE or 3D gradient	Slice thickness 4–5 mmNo slice gap	Axial—suspicious areas *
Matrix size—Minimum 256 (512 is desirable for better resolution).			
**Optional Sequences**			
**Sequence**	**Technique**	**Parameters**	**Plane**
T_2_W	2D SE/TSE	-	Sagittal whole spine
T_2_W	2D SE/TSE	-	Axial—suspicious areas
Heavily weighted T_2_W	2D or 3D CISS/bFFE/FIESTA **	-	Sagittal ± axial
